# The Chain-Mediating Effects of Mindfulness and Sense of Control on Corporate Employees’ Mental Health Problems

**DOI:** 10.3390/bs14080654

**Published:** 2024-07-29

**Authors:** Xiaoran Li, Xiaoli Ni, Juguo Zhang

**Affiliations:** 1 School of Public Administration, Gansu University of Political Science and Law, Lanzhou 730070, China; 16conquer@163.com; 2 Institute of Social Psychology, School of Humanities and Social Sciences, Xi’an Jiaotong University, Xi’an 710049, China; nixiaolipsy@163.com

**Keywords:** work–family conflict, mindfulness, sense of control, mental health, corporate employees

## Abstract

Based on the chain-mediating role of mindfulness and sense of control, this study examines the mediating role of mindfulness and sense of control on employees’ mental health. A total of 720 questionnaires were collected from employees of select enterprises and institutions in China; 53 invalid questionnaires were excluded, with a response rate of 93%, leaving 667 employees as the study sample (average age = 38 years, 71.8% female). The study findings show that: (1) Work–family conflict had a significant positive correlation with mental health problems and a significant negative correlation with mindfulness and sense of control. (2) The influence on the mental health state was due to the mediating effect of mindfulness, sense of control, and the chain-mediating effect of mindfulness and sense of control. The study adopted self-report scales for measuring mindfulness and a sense of control; therefore, further experimental methods must be included in the future to explore these results. This study shows that mindfulness and sense of control can reduce the impact of work–family conflict on mental health problems. Additionally, the chain-mediating effect of mindfulness and sense of control plays an important role in mental health problems.

## 1. Introduction

Mental health has become a public health concern worldwide. These concerns are increasing among young people [1]. Individuals with mental health problems account for nearly 63% of suicide deaths in China. Furthermore, as China modernizes, people are facing severe mental health challenges [2]. Studies have shown that mental health is strongly linked to personal well-being and social harmony [3]. It is helpful to view these mental health problems in the framework proposed by American sociologist Dennis Wrong. He coined the term the “Oversocialized Conception of Man” to indicate that people are conditioned by society to contribute something. Every individual faces the problem that the differential impact of socialization processes conflict with each other. There may also be conflicts between the different roles a person plays in modern society. Therefore, individuals must adjust their roles and deal with conflicts that arise frequently in new situations [4]. For employees, work and family are two inseparable aspects of life; yet, they may generate enormous stress and conflict. These experiences have a key impact on an individual’s mental health.

### 1.1. Work–Family Conflict and Mental Health State

Mental health refers to a state in which an individual has rational cognition, emotional stability, appropriate behavior, and harmonious interpersonal relationships and adapts to changes in the immediate environment during the process of growth and development [5]. However, complex organizational and interpersonal relationships drive adults to deal with the demands of high work pressure and performance, which leads to a variety of psychological problems. Examining somatic symptoms (e.g., headache), anxiety/insomnia, social dysfunction (e.g., social withdrawal), and depression can help to clarify an individual’s mental health status [6].

Work plays an essential role in life. Individuals strive to achieve occupational prestige and respect [7]. Therefore, a positive working environment has benefits contributing to overall personal satisfaction. However, the pressure of juggling family and work may lead to role and interpersonal conflicts [8]. Research shows that work–family conflict adversely impacts employees’ work and home life, general wellness, and other variables related to happiness [9,10].

Work–family conflict is mainly based on role theory [11]. When an individual plays multiple, overlapping, and incompatible roles, the pressure generated by the role conflict causes psychological tension [12]. Employees often struggle to cope with work and family demands [11]. Different social roles create various levels of pressure and burden on individuals. Here, inter-role conflict outlines that “the role pressure of a member from one organization conflicts with that member’s role pressure from the other organization” [13]. Work-to-family (WFC) and family-to-work conflict (FWC) reflect the degree of incompatibility between roles in the work and family domains; that is, “participation in the work (family) role is made more difficult by virtue of participation in the family (work) role” [13]. The excessive demands of the work (family) role make it difficult to fulfill the role of family requirements (work). Therefore, the pressure between work and family leads to work–family conflict. Work can interfere with family life, and family life can also interfere with work. The “Dark Side of Work–Family Conflict” model shows that several studies have confirmed the negative relationship between work–family conflict and health, and individuals who feel a greater work–family conflict will experience more health problems [14].

Frone et al. [15] proposed a comprehensive model of work–family conflict. They found that work interferes with family owing to work pressure and commitment, leading to family conflicts. Moreover, family stress and commitment could result in family interfering with work, leading to work distress. As a general mechanism, the cross-domain model provides a theoretical basis for work–family conflict and health-related problems in China. Very often, people find it difficult to fulfill the requirements of one role owing to obstacles from the other. Therefore, they experience greater psychological pain owing to the difficulty in meeting role demands [15,16].

Goode [17] proposed the theory of role strain. Individuals confronting different requirements for each role but having limited resources of time, energy, and attention to perform their duties also face an increasing possibility of conflict. Work and family are not fully compartmentalized. Thus, there is severe divergence and conflict generated by competing demands [18]. When work interferes with household life, it is difficult to meet family demands; conversely, when family interferes with work, it becomes difficult for the individual to fulfill workplace obligations. This conflict increases psychological pressure, inducing stress and health problems [13,15].

Work and family are two important stages in life and achieving a balance between the two is vital. A harmonious development between the two can improve an individual’s quality of work and life and also improve the relationship between individuals and organizations, family members and peers, and enhance the overall well-being of individuals and families [19].

An increasing number of people are aware of the relationship between family and work [9]. Conflict between the two is often related to factors such as job burnout, life dissatisfaction, or other psychological distresses (such as depression) [13,15]. Previous studies showed that psychologists, nurses, social workers, and others working in hospitals or similar environments are often at risk of burnout, diminished subjective well-being, or poor mental health [20,21]. A meta-analysis showed that both work-to-family conflict (WFC) and family-to-work conflict (FWC) are associated with physical and mental health problems, such as depression and unhealthy physical conditions [9]. Individuals with high levels of work–family conflict report depressive symptoms [22,23] and have poorer general health outcomes [16]. Thus, work–family conflict has a key impact on an individual’s mental health outcomes. Therefore, we propose the following research hypotheses:

**H1a.**  *Work-to-family conflict (WFC) is associated with mental health problems.*

**H1b.**  *Family-to-work conflict (FWC) is associated with mental health problems.*

### 1.2. Mindfulness and Mental Health

Mindfulness is a form of meditation that derives from Buddhist meditation techniques. It involves cultivating a full, direct, and active awareness of experienced spiritual phenomena that are maintained from one moment to the next [24]. An individual can observe all the phenomena (thoughts, emotions, and feelings) in an open and accepting manner without judging his or her own experiences [25,26,27].

Mindfulness is an attribute of consciousness, and its relationship has also been widely observed [28]. Mindfulness is often conceptualized as a character trait that is also observed in individuals who have not accepted mindfulness training [29]. Kabat-Zinn [30] proposed that mindfulness is a method of purposefully and non-judgmentally focusing on the present. Mindfulness comprises three core components: (1) being purposeful or intentional, (2) focusing on moment-by-moment experience, and (3) having a non-judgmental attitude [31,32,33]. Mindfulness separates the individual from unconscious thoughts, behaviors, and unhealthy behavioral patterns, and it fosters conscious and self-approved behavioral regulation. It has long been associated with enhanced well-being.

Clinical psychologists combine mindfulness-based interventions with mental health treatment, arguing that the former can help individuals to recognize and regulate maladaptive thoughts, emotional responses, and automatic behaviors underlying various mental health problems [32]. It is also observed that mindfulness means “waking up” from a “corpse-like” unconscious sleep, which helps individuals to observe and experience their present state. In addition to targeting depression and mood disorders, mindfulness-based interventions are effective in helping to treat mental health problems and physical disorders, including anxiety, anger, and psychiatric disorders, and in improving cognitive functioning and task performance in adults [31,34,35]. Ryan and Deci [36] demonstrated that mindfulness is positively associated with several aspects of mental health, such as higher levels of adaptive emotional regulation, vitality, positive influence, and life satisfaction. Furthermore, it can reduce the occurrence of psychiatric disorders and their negative impact on people [27,37,38,39]. Brown and Ryan [29] demonstrated that there is a significant correlation between mindfulness and well-being, and mindfulness is associated with low levels of neuroticism, anxiety, depression, and negative affect.

Mindfulness-based interventions can significantly reduce stress levels, improve mood [40], and predict negative emotions [41]. Mindfulness has a significant negative correlation with three aspects of the mental health well-being of healthcare professionals [42]. Thus, mindfulness-based interventions can effectively alleviate the occurrence of individual mental health problems.

**H2.**  *Mindfulness reduces the occurrence of mental health problems.*

### 1.3. Sense of Control and Mental Health

In recent years, research on mental health has shown that mental health is the result of the combined effect of physiological, psychological, and social factors. However, given a common stimulus, the severity of mental health damage in individuals varies and is closely related to factors such as individual coping strategy, individual characteristics, and psychological control [43].

Sense of control refers to the extent to which individuals believe they have personal rights and control over their lives and environment [44], and the perceptions and evaluations they have when they believe that their abilities, efforts, and other internal factors affect and determine the development process and changes in external things [45]. Burger and Arkin [46] found that individual perceptions of control or predictability may be sufficient to alleviate feelings of helplessness, and perceptions of control or predictability over adverse events may alleviate learned helplessness. Several studies have shown a correlation between sense of control and physical and mental health [47]. It is believed that sense of control helps people to positively adapt to stressful life situations and promotes physical health and emotional wellness [48,49]. People with a high sense of control act in a healthier manner when they believe that what they do will lead to a difference in outcomes [47]. Broadly, as most objective conditions cannot be changed, people can also modify their individual experience of the objective environment through cognitive means, such as maintaining an optimistic attitude or diverting attention from such experiences, thereby minimizing the feeling of lack of control [50]. Thompson and Prottas [51] found that perceived control had a mediating effect on processing work–family conflict, and control played an important role in an individual’s life.

Beutell and Schneer [52] found that sense of control mediated the relationship between work–family conflict and life satisfaction. Psychologists are concerned about who determines an individual’s behavior and the extent to which individuals can control and influence their living environment and be responsible for their behavior. A lack of sense of control can lead to behavioral problems, such as withdrawal and passive compliance, which are related to the persistence of anxiety, including social anxiety. Thus, sense of control plays a fundamental role in the formation and persistence of social anxiety. For example, sense of control mediates the relationship between safety behaviors, social environment, and social anxiety [53,54]. Jang et al. [55] studied the relationship between perceived discrimination and mental health and found that sense of control plays an important role in mediating and moderating the relationship between perceived discrimination and mental health. In a study on the mental health of armed police officers, Pei et al. [56] found that sense of control had a direct positive impact on armed police officers’ mental health. Their results showed that the higher an individual’s sense of control, the better their mental wellness. In a study on self-focused attention and social anxiety, Huang et al. [57] found that sense of control mediated the relationship between self-awareness and social anxiety. Neupert et al. [58] reported that lower levels of personal control increase sadness and stress, leading to physical health problems. Examining the relationship between life events and depression in elderly persons in Hong Kong and China, Chou and Chi [59] found that sense of control played a mediating role between stressful life events and depression. Additionally, it regulated the occurrence of depression in the elderly. Furthermore, sense of personal control was found to play a partial mediating role in the relationship between psychological distress and reported discrimination experiences [60].

**H3.**  *Sense of control reduces the occurrence of mental health problems.*

### 1.4. Work–Family Conflict, Mindfulness, Sense of Control, and Mental Health

According to role balance theory, balancing roles positively is equivalent to treating each role with a focused and careful attitude. Mindfulness is being alert about the present moment that enables individuals to fully engage in each role with care and focus, as this is also beneficial to role conflict, and mindfulness can improve self-regulation [29,61]. Studies on mindfulness have shown that, compared to people with low scores in mindfulness, individuals with higher levels of mindfulness report less stress, anxiety, and depression symptoms and are more energetic and satisfied with their lives. By contrast, higher levels of mindfulness are associated with a better ability to self-regulate the ongoing internal emotional experience [62]. Behavioral and social environmental factors related to social anxiety can change an individual’s sense of control, which affects an individual’s social anxiety. Specifically, if individuals use safety behaviors, they can increase their sense of control, thereby reducing anxiety. Conversely, adverse or negative thinking reduces an individual’s sense of control, increasing social anxiety [63].

The impact of work–family conflict on mental health is also affected by self-awareness and sense of control. Therefore, mindfulness and sense of control are effective neutralizers. Studies on mindfulness-based interventions show that patients with cancer receiving mindfulness-based training believe they are in better control of their health and the external environment [64]. Gootjes and Rassin [65] reported that mindfulness is related to attention control in daily life. The higher the level of mindfulness, the stronger the perceived thought control. Examining the impact of work–family conflict on sleep quality among Chinese nurses, Liu et al. [66] found that mindfulness played a mediating role in the relationship between work–family conflict and nurses’ sleep quality. Thus, a lower level of mindfulness was associated with poor sleep quality.

**H4.**  *Mindfulness and sense of control mediate the relationship between work–family conflict and mental health problems.*

### 1.5. This Study

Studies show that work–family conflict seriously impacts an individual’s mental health, thereby leading to severe physical and psychological problems. Several studies have explored the impact of work environment, time, job burnout, and other factors on mental health, or simply focused on the impact of work–family conflict on mental health through sense of control, and rarely included mindfulness and sense of control in the research model concurrently. This research focuses on the mental health status of enterprise employees in western China. The main innovation points of this research are as follows: First, the subjects of this study have certain characteristics; here, the mental health status of employees belonging to enterprises and institutions in Northwest China is not common to that in previous studies. Second, the influencing factors selected in this study, including the mediating role of mindfulness and sense of control, have not been featured in existing studies on the mental health status of this group, which is somewhat novel. Furthermore, this study paves the way for some experimental studies to be carried out in follow-up research to enrich the results of this research. Thus, to expand the literature, we examined the impact of individual mindfulness and sense of control on the impact of work–family conflict on mental health to directly mitigate the psychological distress caused by the conflict from the individual itself and reduce the occurrence of mental health problems.

### 1.6. Model Assumptions

**Figure 1:**
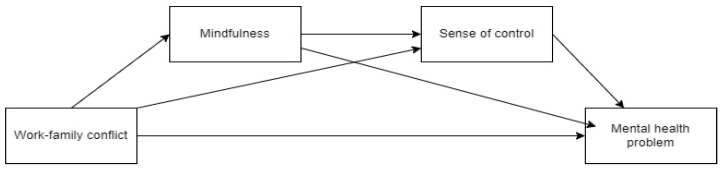
Model diagram.

Based on the above research content, the research model of this paper is shown in Figure 1. Work-family conflict has a direct impact on mental health problems. Meanwhile, work-family conflict has an impact on mental health problems through the chain mediation of mindfulness and sense of control. This paper will further verify the above hypotheses.

## 2. Methods

### 2.1. Participants

This study used random sampling to collect a total of 720 questionnaires from employees of select enterprises and institutions in China’s Shaanxi Province; 53 invalid questionnaires were excluded (valid response rate of 93%). The average participant age was 38 years (*SD* = 9.39, range = 21–64 years), and 71.8% were women.

### 2.2. Research Tools

#### 2.2.1. Work–Family Conflict Scale

The Work–Family Conflict Scale has two dimensions: Work-to-Family Conflict (WFC) and Family-to-Work Conflict (FWC) [18]. Furthermore, the scale has 10 items: five items for each dimension. It is scored on a five-point Likert scale (1 = strongly disagree to 5 = strongly agree). Higher scores indicate more serious conflict. The Cronbach’s α for the WFC scale is 0.89. The Cronbach’s α for the FWC scale is 0.87.

#### 2.2.2. Mindful Attention Awareness Scale (MAAS)

The MAAS, revised by Deng et al. [67], has 15 items and is scored on a six-point scale (1 = almost never to 6 = almost always). The scores for all the items are reversed. Higher scores reflect greater mindfulness. The Cronbach’s α for the MAAS is 0.92.

#### 2.2.3. Sense of Control Scale

The 12-item Sense of Control Scale [44] comprises two dimensions: personal mastery and perceived constraints. Eight items concerning perceived constraints are scored in reverse on a seven-point scale (1 = strongly disagree to 7 = strongly agree). The higher the score, the higher the level of control. Cronbach’s α is 0.71.

#### 2.2.4. Brief Symptom Inventory-18 (BSI-18)

The BSI-18 [68] has 18 items scored on a five-point Likert scale (1 = not at all to 5 = extremely). The scale has three dimensions: somatization, depression, and anxiety. Somatization is caused by the dysfunction of the physical body, manifested in the form of distress to cardiovascular, gastrointestinal, and other physiological systems with strong autonomic regulation. Depression is dissatisfaction and an irritable mood, such as self-deprecation, anhedonia, loss of hope, and suicidal thoughts. Anxiety symptoms include nervousness, jitteriness, restless movement, worry, and panic attacks. The total score of the scale is also known as the Global Severity Index (GSI). Higher scores indicate poor mental health and severe symptoms. Cronbach’s α values for each subscale (somatization, depression, and anxiety) of the BSI-18 and GSI are 0.94, 0.81, 0.86, and 0.90, respectively (Appendix A).

### 2.3. Data Processing

SPSS 26.0 and the SPSS macro program PROCESS were used for data analysis. PROCESS is designed to evaluate and analyze complex models; therefore, we used it to analyze the mediating effects of mindfulness and sense of control on the relationship between work and family stressors and mental health wellness through multiple linear regression analysis [69].

## 3. Results

### 3.1. Testing for Common Method Bias

Harman’s single-factor test was performed to reduce common methods bias. The test results show that the initial eigenvalues of the nine factors were all greater than one, and a single factor explanation was much lower than 40% of the total variance (i.e., 28.24%). Therefore, the data were reliable with no common method bias [70].

### 3.2. Correlation Analysis

Descriptive statistics and correlation analysis results are shown in Table 1. The results show that WFC correlated positively with anxiety, depression, somatization, and the BSI-GSI. Furthermore, WFC correlated negatively with sense of control and mindfulness. FWC correlated positively with anxiety, depression, somatization, and BSI-GSI and correlated negatively with sense of control and mindfulness. Sense of control correlated negatively with anxiety, depression, somatization, and BSI-GSI and correlated positively with mindfulness.

### 3.3. Analysis of Chain Mediating Effects

The SPSS macro, compiled by Hayes [69], was applied. The mediating effects of mindfulness and sense of control on the relationship between WFC and the mental health of employees were analyzed after controlling for age, sex, marital status, and educational background. The regression analysis (Table 2) shows that WFC directly predicted the mental health status (BSI-GSI) of employees (β = 0.18, *p* < 0.01), and WFC directly and negatively predicted mindfulness (β = − 0.98, *p* < 0.001) and sense of control (β = − 0.46, *p* < 0.001); mindfulness directly and positively predicted sense of control (β = 0.36, *p* < 0.001); and mindfulness and sense of control negatively predicted employees’ mental health status (BSI-GSI) (β = − 0.28, *p* < 0.001; β = − 0.17, *p* < 0.001).

**Figure 2:**
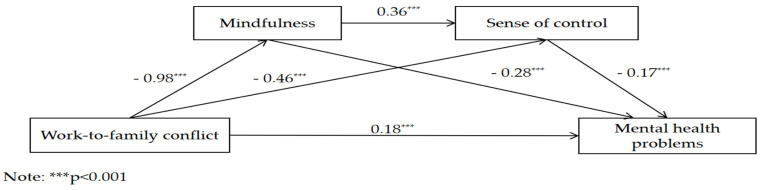
The chain-mediating effects of mindfulness and sense of control.

The results of the analysis of mediating effects (Table 3, Figure 2) show that mindfulness and sense of control played partial mediating roles in the relationship between WFC and mental health, with a mediating effect of 0.41. Specifically, the mediating effects comprised the indirect effects produced by three pathways: indirect effect 1 (0.27) produced through the pathway of WFC → mindfulness → mental health; indirect effect 2 (0.06) produced through the pathway of WFC → mindfulness → sense of control → mental health; and indirect effect 3 (0.08) produced through the pathway of WFC → sense of control → mental health. Table 3 shows that the three indirect effects account for 46.31%, 10.33%, and 13.46% of the total effects, respectively. None of their 95% bootstrap confidence intervals contained 0, indicating that all three indirect effects attained a significant level.

The mediating effects of mindfulness and sense of control on the relationship between FWC and the mental health problems of employees were analyzed. A regression analysis (Table 4) shows that FWC significantly and directly predicted employees’ mental health status (BSI-GSI) (β = 0.16, *p* < 0.01), and FWC directly and negatively predicted mindfulness (β = −0.93, *p* < 0.001) and sense of control (β = −0.54, *p* < 0.001); mindfulness directly and positively predicted sense of control (β = 0.39, *p* < 0.001); and mindfulness and sense of control negatively predicted the mental health status (BSI-GSI) of employees (β = −0.29, *p* < 0.001; β = −0.18, *p* < 0.001).

**Figure 3:**
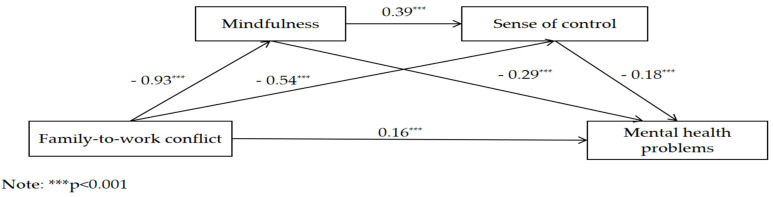
The chain-mediating effects of mindfulness and sense of control.

The results of the analysis of the mediating effects show (Table 5, Figure 3) that mindfulness and sense of control played a partial mediating role in the relationship between FWC and mental health, with a mediating effect of 0.43. Specifically, the mediating effects consisted of indirect effects produced by three pathways: indirect effect 1 (0.27) produced through the pathway of FWC → mindfulness → mental health; indirect effect 2 (0.06) produced through the pathway of FWC → mindfulness → sense of control → mental health; indirect effect 3 (0.10) produced through the pathway of FWC → sense of control → mental health. Table 3 shows that the three indirect effects account for 46%, 18.80%, and 16.30% of the total effects, respectively. None of the 95% bootstrap confidence intervals contained 0, indicating that all three indirect effects reached a significant level.

## 4. Discussion

### 4.1. Correlation Analysis

WFC was significantly and negatively correlated with anxiety, depression, and somatization symptoms, indicating a high correlation between WFC and psychological stress, anxiety, and depression [71]. Bowlin and Baer [72] demonstrated that mindfulness correlates positively with self-control and negatively with psychological symptoms. Individuals experiencing high levels of self-control and mindfulness present lower levels of overall distress.

### 4.2. Mediating Effects Analysis

These results show that mediating effects occurred through three indirect pathways: the independent effect of mindfulness, the independent effect of sense of control, and the combined effect of mindfulness and sense of control.

(1) Independent effect of mindfulness

Zhang et al. [22,23] investigated the work–family conflict and mental health status of nurses and found that increases in work–family conflict reduce mental health scores. Trait mindfulness is significantly and negatively correlated with both WFC and FWC [61].

Employees generally experience severe work pressure and are expected to complete the given assignment and balance the pressure of the various roles in family and society. Therefore, most people experience severe psychological stress, which eventually leads to mental health problems. Investigating the mental health of five types of occupational population in Beijing, Zhao et al. [73] found that anxiety and depression are relatively serious among employees, and this result is consistent with our results. In this study, work–family conflict affected mental health and was manifested in the form of physical symptoms, such as depression and anxiety, and seriously affected the physical and mental wellness of people. Similarly, individuals’ mindfulness and sense of control played a strong mediating role, which effectively alleviated the occurrence of mental illness caused by the conflict between work and family roles, largely mitigating psychological problems. Therefore, mindfulness and sense of control have protective effects on the mental health of adults.

There are several existing studies on mindfulness. Brown and Ryan [29] consider mindfulness to be a witness or observational stance on the current emotions and other psychological experiences, and the results of mindfulness promote a balanced or smooth emotional life. Individuals with high levels of mindfulness are more aware of their own experiences (physical feelings, thoughts, and emotions) [30]. Individuals with an internal locus of control believe they have more control over the outcome of life events and are more proactive in coping with stress [74]. Mindfulness-based training enables people to focus on their current work and family experiences, thereby reducing stress from negative emotions and role conflict and improving mental health. This result is consistent with the findings of this study that the impact of work–family conflict on mental health could be influenced by individuals’ mindfulness. The higher an individual’s level of mindfulness, the more effective it is to reduce the serious impact of work–family conflict on mental health. Therefore, improving an individual’s level of mindfulness can effectively improve mental health wellness.

(2) Independent effect of sense of control

Existing studies have shown that the ability to process one’s thoughts and emotions successfully contributes greatly toward healthy mental functioning [65]. This study found that work–family conflict can directly predict sense of control, further indicating that an individual’s sense of control can effectively reduce the impact of work–family conflict on their mental health and effectively improve their mental health outcomes. Rodin [47] proposed that individuals with a high level of sense of control must adopt better health-promoting behaviors and avoid health-damaging activities. WFC is a key indicator that lowers mental health [28,75]. Therefore, improving an individual’s sense of control has greater significance for mitigating the impact of work–family conflict on mental health.

(3) Combined effect of mindfulness and sense of control

Work–family conflict could also affect individuals’ mental health through the chain-mediating effect of mindfulness and sense of control. The chain-mediating effect effectively reveals how work–family conflict affects individuals’ mental health through the independent and combined effect of the two mediating variables of mindfulness and sense of control, thus illustrating in-depth the mechanism of the impact of WFC on mental health.

## 5. Conclusions

This study confirms that work–family conflict can significantly impact an individual’s mental health problems and promotes research on the effect of mindfulness and sense of control on mental health problems. Furthermore, these results provide valuable guidance for future research related to employees’ mental health problems. On the one hand, individuals can reduce the occurrence of mental health problems by improving mindfulness; on the other, mindfulness has a significant inhibitory effect by reducing the impact of work–family conflict on the mental health of employees by improving an individual’s mindfulness and sense of control. Based on the analysis of factors affecting the mental health of employees, this study found that the continuous improvement in individual mindfulness and the improvement in sense of control can effectively reduce the occurrence of psychological problems. Therefore, leaders in enterprises should train and guide employees to conduct mindfulness training and carry out a series of psychological empowerment activities, which are conducive to the improvement in employees’ mental health. At the same time, experimental methods should be added in future research to explore the impact of mindfulness training on the occurrence of psychological problems, such as depression, among employees.

## Figures and Tables

**Table 1:** Descriptive statistics and bivariate correlations (*n* = 667).

	Mean	SD	1	2	3	4	5	6	7	8
1. WFC	13.75	5.30	1							
2. FWC	9.38	4.30	0.48 **	1						
3. Control	55.19	11.40	−0.40 **	−0.35 **	1					
4. Anxiety	8.20	3.58	0.31 **	0.25 **	−0.40 **	1				
5. Depression	8.52	3.58	0.37 **	0.28 **	−0.48 **	0.82 **	1			
6. Somatization	7.70	2.66	0.30 **	0.26 **	−0.36 **	0.69 **	0.65 **	1		
7. Mindfulness	65.80	12.43	−0.41 **	−0.32 **	0.49 **	−0.50 **	−0.53 **	−0.45 **	1	
8. GSI	24.40	8.90	0.36 **	0.29 **	−0.46 **	0.94 **	0.93 **	0.84 **	−0.55 **	1

Note. SD = standard deviation. WFC = work-to-family conflict. FWC = family-to-work conflict. GSI = Global Severity Index. ** *p* < 0.01

**Table 2:** Multiple linear regression analysis results for testing the mediating role of mindfulness and control in the relationship between work-to-family conflict and mental health (*n* = 667).

Predictor Variable	Outcome Variable	*R*	*R* ^2^	*F*	*β*	*t*	Boot LLCI	Boot ULCI
WFC	Mindfulness	0.44	0.20	19.90	−0.98	−11.78 ***	−1.14	−0.82
WFC	Control	0.54	0.29	30.20	−0.46	−5.90 ***	−0.62	−0.31
Mindfulness					0.36	10.85 ***	0.30	0.43
WFC	GSI	0.61	0.37	38.24	0.18	2.96 **	0.60	0.29
Mindfulness					−0.28	−10.39 ***	−0.33	−0.23
Control					−0.17	−5.95 ***	−2.23	−0.12

Note. Boot LLCI and Boot ULCI are 95% confidence interval lower and 95% confidence interval upper calculated by the bias-corrected percentile bootstrap method for testing indirect effects. The 95% confidence intervals do not overlap with zero. GSI = Global Severity Index. WFC = work-to-family conflict. ** *p* < 0.01, *** *p* < 0.001.

**Table 3:** Indirect effects of mindfulness and control (*n =* 667).

	Effect	Boot SE	Boot LLCI	Boot ULCI	Ratio of Indirect to Total Effect
Total indirect effect	0.43	0.06	0.31	0.54	70.88%
Indirect effect 1	0.28	0.05	0.19	0.38	45.86%
Indirect effect 2	0.07	0.01	0.04	0.10	10.82%
Indirect effect 3	0.09	0.02	0.05	0.13	14.20%

Notes. Indirect effect 1 was Work-to-family conflict → Mindfulness → Mental health. Indirect effect 2 was Work-to-family conflict → Control → Mental health. Indirect effect 3 was Work-to-family conflict → Mindfulness → Control → Mental health. Boot SE, Boot LLCI, and Boot ULCI are estimated standard errors, 95% confidence interval lower, and 95% confidence interval upper, respectively, through the bias-corrected percentile bootstrap method, used for testing indirect effects. The 95% confidence intervals do not overlap with zero.

**Table 4:** Multiple linear regression analysis results for testing the mediating role of mindfulness and control in the relationship between family-to-work conflict and mental health.

Predictor Variable	Outcome Variable	*R*	*R* ^2^	*F*	*β*	*t*	Boot LLCI	Boot ULCI
FWC	Mindfulness	0.35	0.13	11.71	−0.93	−8.66 ***	−1.14	−0.72
FWC	Control	0.54	0.29	30.04	−0.54	−5.80 ***	−0.73	−0.36
Mindfulness					0.39	12.02 ***	0.32	0.45
FWC	GSI	0.60	0.37	37.65	0.16	2.23 **	0.02	0.30
Mindfulness					−0.29	−11.07 ***	−0.34	−0.24
Control					−0.18	−6.10 ***	−2.24	−0.12

Notes. Boot LLCI and Boot ULCI are 95% confidence interval lower and 95% confidence interval upper calculated by the bias-corrected percentile bootstrap method for testing indirect effects. The 95% confidence intervals do not overlap with zero. GSI = Global Severity Index. FWC = family-to-work conflict. ** *p* < 0.01, *** *p* < 0.001.

**Table 5:** Indirect effect of mindfulness and control (*n =* 667).

	Effect	Boot SE	Boot LLCI	Boot ULCI	Ratio of Indirect to Total Effect
Total indirect effect	0.45	0.06	0.31	0.57	74.61%
Indirect effect 1	0.28	0.05	0.18	0.40	46.37%
Indirect effect 2	0.07	0.01	0.04	0.10	11.54%
Indirect effect 3	0.10	0.02	0.06	0.16	16.70%

Notes. Indirect effect 1 was Family-to-work conflict → Mindfulness → Mental health. Indirect effect 2 was Family-to-work conflict → Control → Mental health. Indirect effect 3 was Family-to-work conflict → Mindfulness → Control → Mental health. Boot SE, Boot LLCI, and Boot ULCI are estimated standard error, 95% confidence interval lower, and 95% confidence interval upper, respectively, through bias-corrected percentile bootstrap method used for testing indirect effects. The 95% confidence intervals do not overlap with zero.

## Data Availability

The data presented in this study are available on request from the corresponding author.

## Appendix A

**Table A1:** Brief symptom inventory-18 (BSI-18).

No	Items	Not at All	Slight	Moderate	Relatively Serious	Extremely
1	Faintness or dizziness	1	2	3	4	5
2	Feeling no interest in things	1	2	3	4	5
3	Nervousness or shakiness inside	1	2	3	4	5
4	Pains in heart or chest	1	2	3	4	5
5	Feeling lonely	1	2	3	4	5
6	Feeling tense or keyed up	1	2	3	4	5
7	Nausea or upset stomach	1	2	3	4	5
